# Journalistic Changes

**Published:** 1883-04

**Authors:** 


Journalistic Changes.—We are pleased to notice some improvements and changes in our journalistic exchanges. *The Medical Record* is enlarged in size, *The Indiana Farmer* has changed from a double sheet to a sixteen-page paper, and *The Nashville Journal of Medicine and Surgery* has increased its reading matter, looking quite attractive in its new form ; *The National Live Stock Journal* appears in a new set of type, and *The Rocky Mountaiu Times* has changed its title to that of *The Denver Medical Times*, Dr. F. Marquand Trask being added tó the editorial staff. *The Chicago Medical Review*, formerly issued semi-monthly, has been consolidated with *Chambers' Medical Review* and is now issued every week simultaneously in Chicago and St. Louis, under the title of *Weekly Medical Review*, and published by J. H. Chambers & Co.

